# SAFlex: A structural alphabet extension to integrate protein structural flexibility and missing data information

**DOI:** 10.1371/journal.pone.0198854

**Published:** 2018-07-05

**Authors:** Ikram Allam, Delphine Flatters, Géraldine Caumes, Leslie Regad, Vincent Delos, Gregory Nuel, Anne-Claude Camproux

**Affiliations:** 1 Molécules thérapeutiques in silico (MTi), INSERM UMR-S973, University Paris Diderot, Paris 7, France; 2 Probability Statistique and Biology (PSB), LPMA laboratory, CNRS INSMI UMR 7599, University Pierre et Marie Curie, Paris 6, France; 3 Mathématiques Appliquées, MAP5 laboratory, CNRS UMR 8145, University Paris Descartes, Paris 5, France; 4 Sorbonne Paris Cité, Paris, France; Universita degli Studi di Roma Tor Vergata, ITALY

## Abstract

In this paper, we describe SAFlex (Structural Alphabet Flexibility), an extension of an existing structural alphabet (HMM-SA), to better explore increasing protein three dimensional structure information by encoding conformations of proteins in case of missing residues or uncertainties. An SA aims to reduce three dimensional conformations of proteins as well as their analysis and comparison complexity by simplifying any conformation in a series of structural letters. Our methodology presents several novelties. Firstly, it can account for the encoding uncertainty by providing a wide range of encoding options: the maximum a posteriori, the marginal posterior distribution, and the effective number of letters at each given position. Secondly, our new algorithm deals with the missing data in the protein structure files (concerning more than 75% of the proteins from the Protein Data Bank) in a rigorous probabilistic framework. Thirdly, SAFlex is able to encode and to build a consensus encoding from different replicates of a single protein such as several homomer chains. This allows localizing structural differences between different chains and detecting structural variability, which is essential for protein flexibility identification. These improvements are illustrated on different proteins, such as the crystal structure of an eukaryotic small heat shock protein. They are promising to explore increasing protein redundancy data and obtain useful quantification of their flexibility.

## Introduction

Over the past two decades, the notion of a structural alphabet (SA) has attracted much attention. SA encodes protein fragments into structural letters (SL). SA encoding plays a key role in compressing the three-dimensional (3D) protein conformations into a one-dimensional (1D) SL representation, thereby allowing for a simplified protein structure analysis [1–5]. This approach also dramatically simplifies the comparison of 3D conformations by using well-known sequence comparison algorithms (ex: local score from Smith and Waterman, [6]) on SL sequences.

Many studies have developed SAs, based on mixture models [7], classification methods such as AutoANN [8], SOM [9] and K-Nearest Neighbor [10]: Structural Building Blocks [11], Protein Blocks [4], SABD [12] and USA [13], M32K25 [14]; and hidden Markov model (HMM): HMM-SA [2, 3, 15]. The choice between these methods and models plays a major part in the construction of an accurate SA. They have been applied in the past to protein structure analysis, including multiple structure alignment, structure mining [16, 17], protein fold classification [18], dynamic molecular analysis [19–21], structure fast comparison [17] and generation of 3D peptide conformations [22, 23]. The SA approach also appears to be promising to characterize structural variability [24, 25], to explore the local backbone deformation involved in protein-protein interactions [26, 27] and to predict local protein flexibility [28, 29].

However, current SAs have not been trained to take into account the wealth of available protein structure data: their uncertainty (ex: missing data) and redundancy (ex: multiple homomers chains). The growth and speed of macromolecular structure determination techniques (protein crystallography and NMR spectroscopy) results in a considerable increase to 3D structures in the Protein Data Bank (PDB, [30, 31]), which currently has more than 130,000 3D protein structures. More than half of PDB structures share at least 95% sequence identity. Even if this redundancy is considered valuable in investigating families of homologous sequences [32, 33], the dominant approach for data mining the PDB considers redundancy as non-informative [34], resulting in an artificial reduction in the variability of the structural space. Yet, protein redundancy analysis is crucial for protein flexibility insight. Proteins are highly flexible macromolecules and their 3D folding and dynamic properties are essential in many biological processes [32, 34]. Integrating PDB redundancy has potential to improve understanding of protein intrinsic flexibility [35]. PDB files include monomers corresponding to individual protein chain but also homomeric complexes formed by the assembly of multiple copies of a single type of polypeptide chain, and heteromic complexes formed from multiple distinct polypeptide chains [36]. Different PDB files can also correspond to a same protein in different conditions, called multi-conformations. For instance, in 2015, the non-redundant snapshot of protein crystal structures contained 7,972 monomers and 9,206 homomers and 2,677 heteromers [37]. The authors concluded, 87% of crystal structures involve only a single type of polypeptide chain, and a slight majority (54%) of these self-assemble into homomers. Thus, it is a very important point to be able to model this multiple chain data.

Another source of uncertainty in PDB data analysis is that most PDB structures have some missing parts which strongly impact the determination of their accurate protein folding and lead to important difficulty for protein structure and function interpretation [38]. This kind of issue is very serious, from missing side chains, entire loop regions, to whole domain. For instance, it was demonstrated in 2007 by [39] that ∼10% of 16,370 PDB X-ray structure files contain regions of more than 30 missing or ambiguous amino acids and ∼40% have missing or ambiguous regions between 10 and 30 amino acids. These missing parts can result from some resolution difficulty or from intrinsic flexibility of proteins [38, 39]. The absence of the coordinates of the alpha-carbon, which makes it possible to connect to the peptide skeleton, poses a serious problem because it effectively prevents knowledge of the secondary structure and of the 3D folding. These missing parts often relate to the ends of the protein or loops that are the most flexible regions of proteins and involved in protein interactions and function. Thus the detection and modeling of these missing data could have a very appealing impact for protein structure analysis. However, they have not been explicitly modeled by different SA approaches.

In this paper, we extend a previously published SA, HMM-SA [3, 15, 40], in SAFlex (Structural Alphabet Flexibility) to encode 3D conformations of proteins in case of missing residues or uncertainties with the aim to better explore increasing protein 3D structure information. HMM-SA was modeled using HMM, which provides a very precise description of protein structures, particularly loop regions [41] known to play important roles in protein function. One major contribution of HMM is that this model implicitly takes the SL sequential connections into account. For example, this markovian modeling allows for efficient extraction of functional motifs [5, 41]. The paper is organized as follows. The “Materials and Methods” section provides descriptions of the improvements based on our HMM modeling. It presents a collection of technical advances including a wide range of encoding options (the maximum a posteriori, the marginal posterior distribution, and the effective number of letters at each given position), the robustness of missing data in the PDB files, and the ability to encode a monomer or an heteromer as well as different replicates of a single protein with multiple chains (homomers) leading to a consensus encoding. This approach is however not yet able to directly take into account protein with multi-conformations (*i.e*. several PDB files). The “Results” section illustrates the application of our new approach on different PDB structures of interest. Finally, the “Conclusion” section summarizes the manuscript and discusses potential development and application of SAFlex for addressing structural biology challenges.

## Materials and methods

In this section, we describe the model used to encode protein structures (PDB files) into SL sequences. To that aim, we introduce an HMM in which the spatial conformation of the protein is the observation and the underlying structural sequence is the hidden part.

### Structural fragments

**Fig 1:**
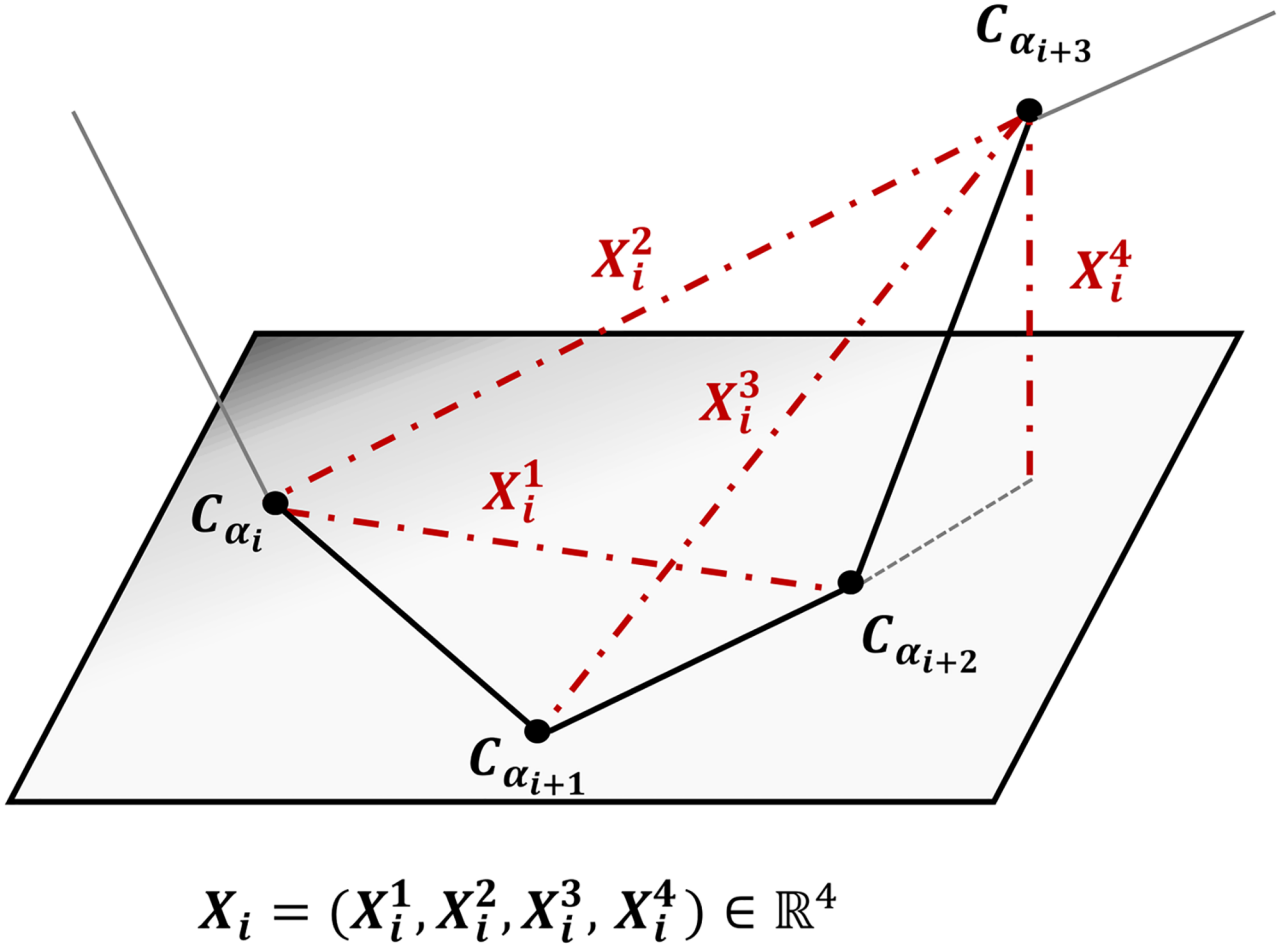
The four descriptors $X_{i}=\left(X_{i}^{1},X_{i}^{2},X_{i}^{3},X_{i}^{4}\right)\in R^{4}$ for the *i*^th^ fragment (alpha carbons $C_{\alpha_{i}}$ to $C_{\alpha_{i+3}}$).

The PDB file input, obtained from the worldwide Protein Data Bank (wwPDB) (http://www.wwpdb.org/) [42, 43], contains the atomic coordinates describing the 3D structure of the protein. Since the original observation (3D structures from PDB files) is highly complex, we start by reducing this complexity to a sequence of numeric descriptors as in the original HMM-SA publications [2, 3]. Starting from the 3D positions of the alpha carbons (denoted a *C*_*α*_), “*fragment*” is a succession of four consecutive *C*_*α*_ and we use the four descriptors in Fig 1. Formally, for the *i*^th^ fragment we have: $X_{i}^{1}=D\left(C_{\alpha_{i}},C_{\alpha_{i+2}}\right)$, $X_{i}^{2}=D\left(C_{\alpha_{i}},C_{\alpha_{i+3}}\right)$, $X_{i}^{3}=D\left(C_{\alpha_{i+1}},C_{\alpha_{i+3}}\right)$, and $X_{i}^{4}=\eta D\left(C_{\alpha_{i+3}},H\right)$ where *D* is the Euclidian distance, *H* is the orthogonal projection of $C_{\alpha_{i+3}}$ on the plane $\left(C_{\alpha_{i}},C_{\alpha_{i+1}},C_{\alpha_{i+2}}\right)$, and where *η* = +1 (resp. −1) if the cross-product of vector $C_{\alpha_{i}}\to C_{\alpha_{i+2}}$ and vector $C_{\alpha_{i}}\to C_{\alpha_{i+1}}$ has the same (resp. opposite) direction than vector $H\to C_{\alpha_{i+3}}$. Since a structural fragment is formed by four consecutive alpha carbons, a sequence of *n*+ 3 alpha carbons will have only a total of *n* fragments with successive fragments overlapping on three alpha carbons. Hence the Fragment *i* corresponds to the four alpha carbons: $C_{\alpha_{i}},C_{\alpha_{i+1}},C_{\alpha_{i+2}},C_{\alpha_{i+3}}$. Of course, numerous other structural alphabets are based on different geometrical descriptors such as angles and torsions descriptors but we can note several studies such as [1, 44–51] focus on geometrical descriptors based on RMSD, on cRMD between alpha carbons, or, like our own descriptors, using Euclidean distances between alpha carbons.

### A hidden markov model

**Fig 2:**
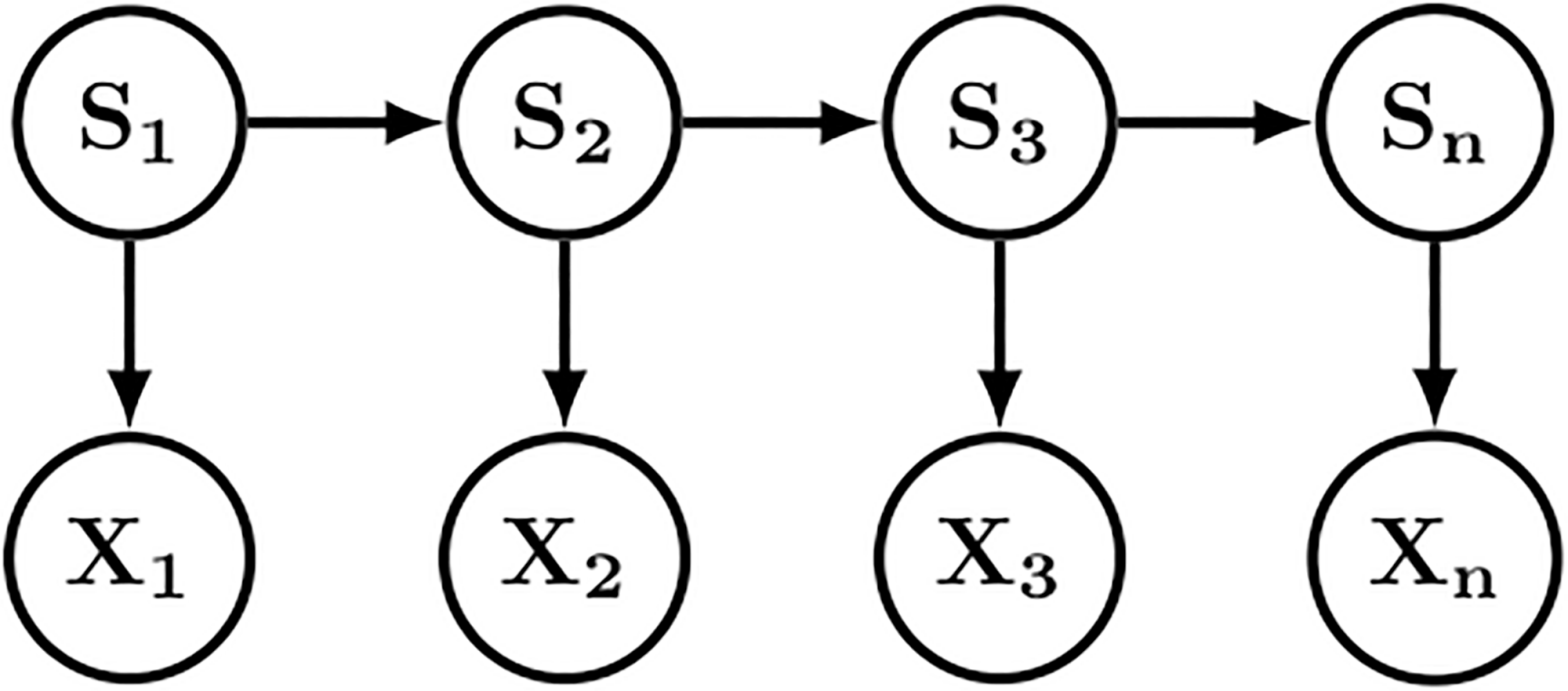
The HMM-SA model. $X_{i}\in R^{4}$ are the fragment descriptors, and *S*_*i*_ ∈ {1, 2, …, m} are the structural letters.

Our idea is to consider the sequence $X_{1:n}\in R^{n\times 4}$ of *n* structural fragments as the observed states of an HMM where the hidden states are the SL *S*_1:*n*_ ∈ {1, …, *m*}^*n*^. A Markov dependency is assumed among the *m* SL. A (conditional) Gaussian model is used for the fragment descriptor distribution. The resulting model, represented in Fig 2, has the following probability distribution:

$$\begin{array}{c} P\left(X_{1:n},S_{1:n}\right)=\underset{\text{Markov}\;\text{part}}{\underset{︸}{P\left(S_{1}\right)\prod_{i=2}^{n}P\left(S_{i}|S_{i-1}\right)}}\times \underset{\text{Emission}\;\text{part}}{\underset{︸}{\prod_{i=1}^{n}P\left(X_{i}|S_{i}\right)}} \end{array}$$ (1)

Assuming that the Markov chain has a uniform starting distribution, a homogeneous transition matrix $\pi \in R^{m\times m}$, and that we denote $e_{i}\left(S_{i}\right)=P\left(X_{i}|S_{i}\right)$ we get the following simplified equation:

$$\begin{array}{c} P\left(X_{1:n},S_{1:n}\right)=\frac{1}{m}\prod_{i=2}^{n}\pi \left(S_{i-1},S_{i}\right)\prod_{i=1}^{n}e_{i}\left(S_{i}\right) \end{array}$$ (2)

For the emission distribution, we simply assume that fragment descriptors are Gaussian distributed, (with a specific mean vector $\mu_{s}\in R^{4}$ and covariance matrix $\Sigma_{s}\in R^{4\times 4}$), for each structural letter *s* ∈ {1, …, *m*} which hence gives:

$$\begin{array}{c} \log e_{i}\left(s\right)=\text{cst.}-\frac{1}{2}\log\det\Sigma_{s}-\frac{1}{2}{\left(X_{i}-\mu_{s}\right)}^{T}\Sigma_{s}^{-1}\left(X_{i}-\mu_{s}\right). \end{array}$$ (3)

One should note that the choice of a uniform starting distribution (rather than an estimation as in the case of original HMM-SA publications [2, 4]) has little impact on the encoding (Markov has a short range dependency), but is quite useful in terms of parsimony, since the resulting model has less parameters to estimate.

### Protein encoding

#### Forward and backward

In order to perform exact inference in hidden Markov models, it is a common practice to introduce the so-called forward and backward quantities. The expression of these quantities along with the mathematical results allows for computing and deriving probabilistic quantities of interest. This can be found in classical reference textbooks (see [52] for example). For completeness, we present a brief form of the forward/backward quantity definition and the key mathematical results.

The Forward and Backward quantities are defined for all *i* = 2 … *n* and for all SL *s* by:

$$\begin{array}{c} \left\{ \begin{array}{c} F_{i}\left(s\right)=\sum_{S_{1:i-1}}P\left(X_{1:i},S_{1:i-1},S_{i}=s\right)=P\left(X_{1:i},S_{i}=s\right)\\ B_{i-1}\left(s\right)=\sum_{S_{i:n}}P\left(X_{1:i},S_{i:n}|S_{i}=s\right)=P\left(X_{1:i}|S_{i}=s\right) \end{array}\right. \end{array}$$ (4)

with *F*_1_(*s*) = *e*_1_(*s*)/*m* and *B*_*n*_(*s*) = 1.

These quantities can be computed recursively through a recursion on *i* = 2 … *n* (resp. *i* = *n* … 2) for forward (resp. backward) given for all SL *r* and *s* by:

$$\begin{array}{c} \left\{ \begin{array}{c} F_{i}\left(s\right)=\sum_{r}\;F_{i-1}\left(r\right)\pi \left(r,s\right)e_{i}\left(s\right)\\ B_{i-1}\left(r\right)=\sum_{s}\;\pi \left(r,s\right)e_{i}\left(s\right)B_{i}\left(s\right) \end{array}\right. \end{array}$$ (5)

#### Marginal posterior distribution

First, we focus on the marginal posterior distribution (POST) which can be immediately derived from the forward/backward quantities:

$$\begin{array}{c} {\text{POST}}_{i}\left(s\right)=P\left(S_{i}=s|X_{1:n}\right)\propto F_{i}\left(s\right)B_{i}\left(s\right)=\frac{F_{i}\left(s\right)B_{i}\left(s\right)}{\sum_{r}F_{i}\left(r\right)B_{i}\left(r\right)}. \end{array}$$ (6)

This POST also can be used to quantify the level of uncertainty of the SL encoding by computing the marginal posterior entropy (ENT) and effective number of SL (NEFF).

$$\begin{array}{c} {\text{ENT}}_{i}=-\sum_{s}{\text{POST}}_{i}\left(s\right)\log{\text{POST}}_{i}\left(s\right)\;\text{and}\;{\text{NEFF}}_{i}=\exp\left({\text{ENT}}_{i}\right) \end{array}$$ (7)

ENT_*i*_ is a measure of disorder for Fragment *i*. If the posterior distribution is a Dirac, ENT_*i*_ = 0, the minimal entropy, if the encoding uncertainty is maximum with POST_*i*_(*s*) = 1/*m*, ENT_*i*_ = log *m* is maximal. The effective number of SL NEFF_*i*_ ∈ [1, *m*] provides a simpler interpretation of the entropy as the effective number of SL acceptable for Fragment *i*.

#### Maximum a posteriori

The task of encoding a 3D structure (sequence of *n* fragments) into a structural sequence can be achieved by computing the Maximum a Posteriori (MAP) defined by:

$$\begin{array}{c} {\text{MAP}}_{1:n}=\arg\max_{S_{1:n}}P\left(S_{1:n}|X_{1:n}\right)=\arg\max_{S_{1:n}}P\left(X_{1:n},S_{1:n}\right). \end{array}$$ (8)

For computing the MAP, we need to introduce the max-forward and max-backward quantities which are defined and recursively computed by simply replacing all ‘∑’ occurrences by ‘max’ in Eqs (4) and (5). Once the max-forward and max-backward quantities are computed, it is possible to obtain the MAP immediately with:

$$\begin{array}{c} {\text{MAP}}_{i}=\arg\max_{s}F_{i}^{\text{max}}\left(s\right)B_{i}^{\text{max}}\left(s\right)\;\;\text{for}\;\text{all}\;i=1\ldots n. \end{array}$$ (9)

Note that MAP_*i*_ is not necessarily equal to arg max_*s*_ POST_*i*_(*s*) since POST is a *marginal* posterior distribution. However, the two quantities are often the same when the posterior distribution is ‘sharp’ enough.

### Missing data

When dealing with 3D proteins structures, it is quite common that some alpha carbon positions are missing, which results in several missing data in fragment descriptors. Therefore it is necessary to deal with these missing data efficiently, which is quite straightforward under the Gaussian assumption. Let the subset of non-missing descriptors for fragment *i* be denoted by *J* ⊂ {1, 2, 3, 4}. *J* = ∅ if all descriptors are missing, and *J* = {1, 2, 3, 4} if none are missing. Then the emission probability of fragment *j* for the structural letter *s* is obtained by considering the restriction $X_{i}\left[J\right]∼N\left(\mu_{s}\left[J\right],\Sigma_{s}\left[J,J\right]\right)$. By convention, *e*_*i*_(*s*) = 1 for all *s* if *J* = ∅ meaning that the totally missing fragment *i* is totally uninformative.

### Multiple chains

**Fig 3:**
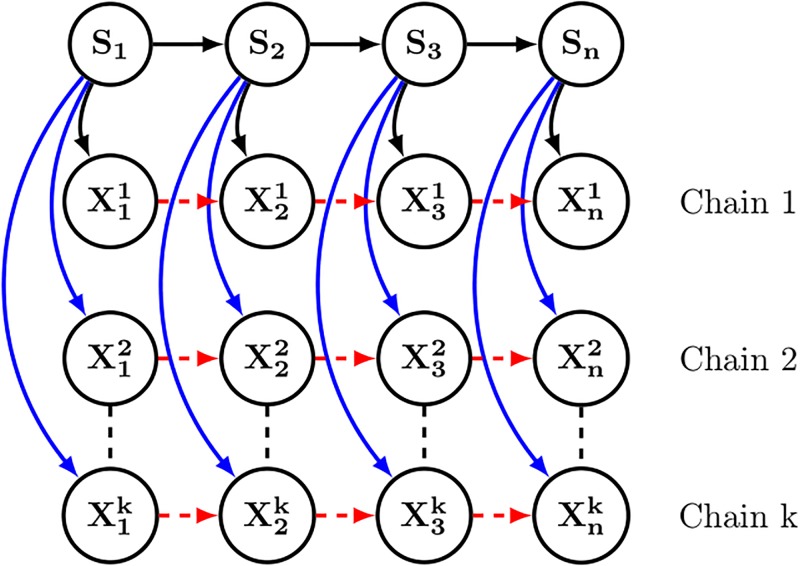
The SAFlex model with k chains.

In the PDB, some proteins are represented several times. They could be simple replicates, or 3D structures in different conditions (e.g. associated or not with various partners, with or without mutated positions). It is obviously possible to perform one encoding for each of these replicates, but there is also another possibility: build a consensus encoding from the whole replicate set. This can be done easily by considering the model in Fig 3 which results in the following likelihood:

$$\begin{array}{c} P\left(X_{1:n}^{1:k},S_{1:n}\right)=\underset{\text{consensus}\;\text{encoding}}{\underset{︸}{P\left(S_{1}\right)\prod_{i=2}^{n}P\left(S_{i}|S_{i-1}\right)}}\times \prod_{j=1}^{k}\underset{\text{emission}\;\text{for}\;\text{Chain}\;j}{\underset{︸}{\prod_{i=1}^{n}P\left(X_{i}^{j}|S_{i}\right)}} \end{array}$$ (10)

The previous encoding algorithm can be adapted easily to this new context by slightly changing the expression of *e*_*i*_(*s*):

$$\begin{array}{c} \log e_{i}\left(s\right)=\text{cst.}-\sum_{j=1}^{k}(\frac{1}{2}\log\det\Sigma_{s}+{\left(X_{i}^{j}-\mu_{s}\right)}^{T}\Sigma_{s}^{-1}\left(X_{i}^{j}-\mu_{s}\right)). \end{array}$$ (11)

Note that missing data can also be easily taken into account in this context. In the particular case when a large portion of data are missing in multiple chains, the model can easily cope with this situation as long as all the chains are properly aligned using a common reference index. In such a situation, a particular position would typically be informative only for a small portion of the chains, which is not a problem for the model.

### SAFlex structural alphabet implementation

An SAFlex preliminary web server including dynamic pages (php/javascript/html/css) is made freely available to the scientific community. The backend was developed in C++ language as a high performance encoding program. All computations (evidence, forward, backward and posterior marginal) are performed in logarithmic scale and thus allow for low probabilities. The SAFlex server is able to complete encoding and indication of data uncertainties in less than one second (in most cases). The SAFlex web server has been successfully tested in the latest version of Chrome, Firefox and Safari. SAFlex is available at the following URL http://saflex.rpbs.univ-paris-diderot.fr/SA-Encoder.php.

## Results

### A new structural alphabet encoding: SAFlex

Here we propose to extend the SA-based approach, HMM-SA ([3, 15]) in SAFlex, to take into account 3D protein flexibility and redundancy as well as missing data. For completness, we briefly recall the HMM-SA construction methodology. The backbone of protein structures was split in overlapping fragments of four residues with each one described by the four descriptors illustrated in Fig 1. HMM-SA was estimated using a collection of non-redundant globular proteins, presenting less than 30% of sequence identity. Only proteins of at least 30 amino acids long, no chain breaks, and obtained by X-ray diffraction with a resolution greater than 2.5 Å were retained. This resulted in a collection of 1,429 protein chains, a total of 336,780 amino acids and 332,493 four-residue fragments. The optimal structural alphabet model was selected by comparing structural alphabets of different number of SL using the Bayesian Information Criterion, which balances the log-likelihood of the model and a penalty term related to the number of parameters of the model and the sample size. HMM-SA results in *m* = 27 SL: four SL specific to helices, five SL specific to strands and the remaining 18 SL that describe loops [15].

**Fig 4:**
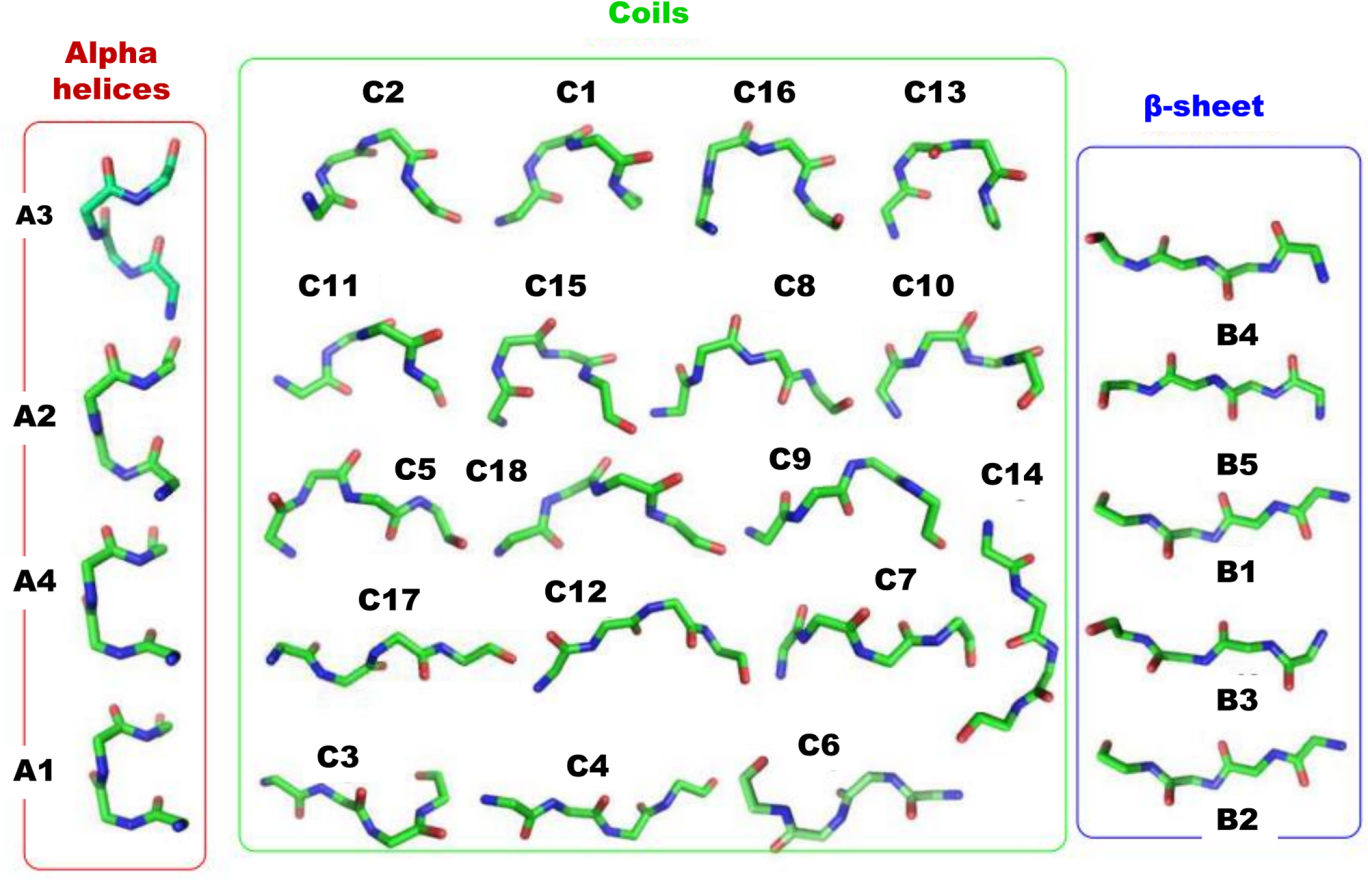
Structural letter prototypes of SAFlex. The 27 representative 3D structural fragments associated with 27 SL of SAFlex. The SL are classified into three groups relative to their secondary structure correspondence: alpha-helices, coils and beta-strands, as described in [3]. The alpha-helices correspond to 4 SL, the coils to 16 SL and beta-strands to 5 SL. Main trajectories between 27 SL of SAFlex.

SAFlex corresponds to 27 HMM-SA SL but in order to improve the interpretability of the 27 SL, a novel nomenclature is applied in SAFlex. This structural letter assignment now depends on the secondary structure type, as identified using the STRIDE software [53] in [3]. Hence, we use only three letters (‘A’,‘B’,‘C’) which refer to the class of SL (*Helices*, *Strands* or *Coils* respectively) as indicated in Table 1. Each letter assignment is followed by a number indicating its frequency in the data set after unsupervised learning. Thus, SL are ranked in descending order according to their frequencies. For example: the letter ‘A1’ is more frequent than the letter ‘A2’. The correspondence with the previous HMM-SA nomenclature is provided in Table 1. SA nomenclature update and corresponding frequency, effective number of acceptable SL in input or output are also indicated. The covariance matrix *Σ* of each SL is provided together with the four descriptors $X=\left(X^{1},X^{2},X^{3},X^{4}\right)\in R^{4}$ in supplementary Data, (S1). For completeness, the SAFlex 27 representative fragments are illustrated in Fig 4.

**Table 1:** SAFlex structural description. Nomenclature correspondence between HMM-SA and SAFlex SL. Frequency corresponds to associated frequency observed in the HMM-SA training data set, NEFF_output (resp. NEFF_input) corresponds to the effective number of acceptable SL in output (resp. input).

	27 structural letters																										
	helices				coils																		strands				
SAFlex	A1	A2	A3	A4	C1	C2	C3	C4	C5	C6	C7	C8	C9	C10	C11	C12	C13	C14	C15	C16	C17	C18	B1	B2	B3	B4	B5
HMM-SA	A	V	W	a	B	Z	P	K	Q	G	S	I	H	D	E	J	U	Y	F	C	R	O	M	L	N	X	T
**Frequency %**	12.6	5.6	5.3	2.6	4.7	4.5	4.4	4.1	4.1	3.4	3.2	2.9	2.7	2.0	2.0	2.0	2.0	2.0	1.9	1.8	1.7	1.5	5.3	5.1	4.9	4.7	3.0
**NEFF_output**	2.4	3.6	3.7	2.8	9.6	11.7	11.8	10.4	10.7	12.6	10.4	9.3	9.6	4.3	12.4	11.7	8.5	15.5	8.9	11.6	11.8	13.0	7.9	10.8	9.6	9.2	7.6
**NEFF_input**	2.3	4.5	3.4	3.0	9.0	12.4	13.3	10.5	12.2	17.5	8.3	7.1	9.7	9.6	11.2	8.8	12.5	10.8	13.5	6.2	12.4	9.8	8.0	10.8	7.0	10.4	7.7

**Fig 5:**
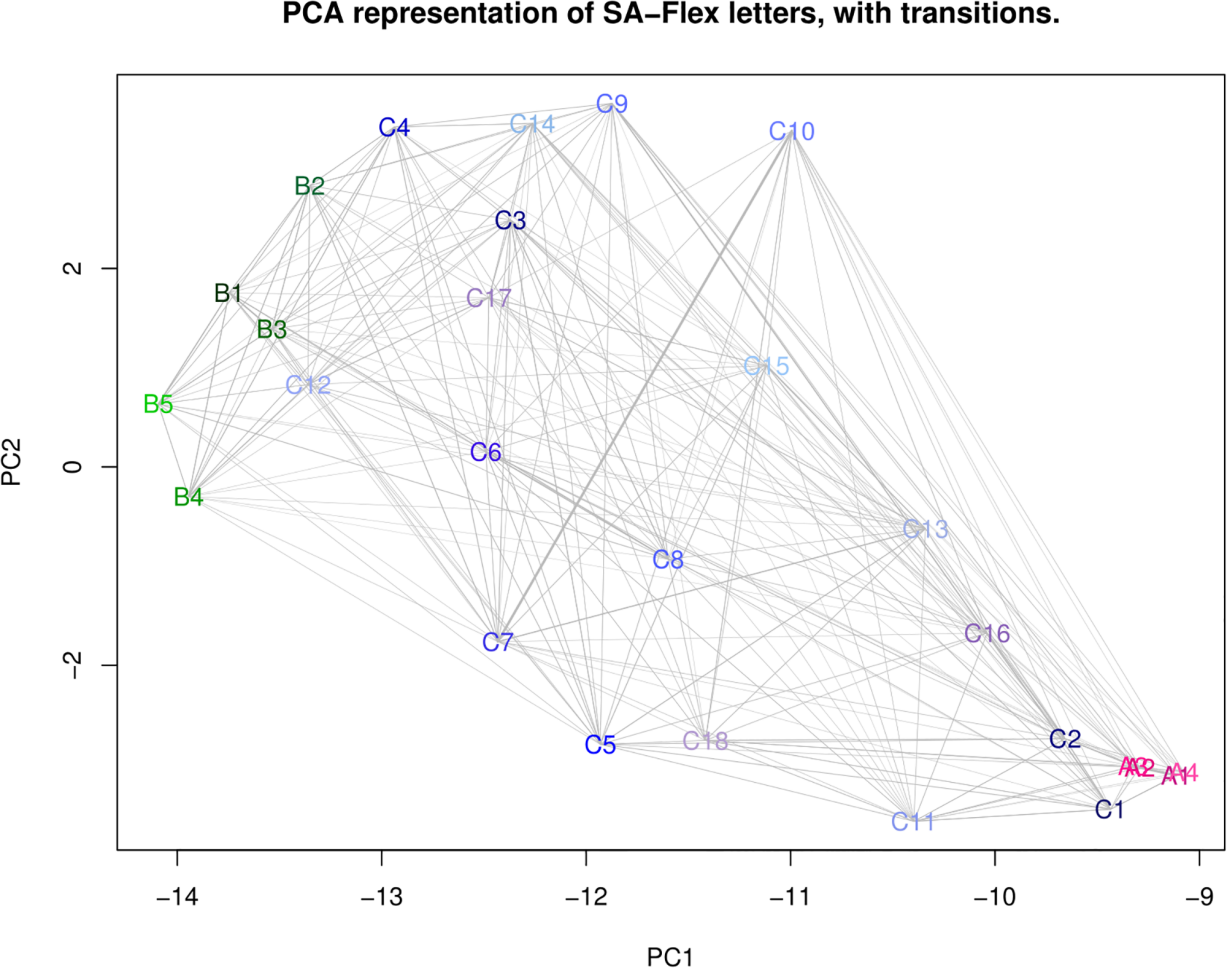
Main Markovian transitions between 27 SL of SAFlex. The SL are projected in the PCA (97.45% of explained variance with the two first axis) space formed by the expected value of the geometrical descriptors of each SL. Orientated Markovian (non reflexive) transitions above 20% probability (resp. between 10% and 20% probability) are represented with a solid (resp. dashed) arrow. There is no direct transition between SL associated with Helices and with Strands.

To illustrate the interrelationship of the 27 SL, Fig 5 provides a graphical representation of the main Markovian transitions. Unsurprisingly, the transition structure is highly asymetrical, and most transitions occur within SL from belonging to the same secondary structure class. Nevertheless, as previously pointed out [15], there are recurrent transitions from secondary structure classes through specific SL but indirect transitions between Helices and Strands. The complete transition matrix of the SA is described in [3].

As explained in the “Materials and Methods” section, our model provides three different encoding information for each chain: 1) the MAP offers the most probable SL sequence fully taking account the complex dependence structure between the SL (including transition probabilities). This is typically the primary encoding used by experimentalists for structure analysis and comparison; 2) in order to account for encoding uncertainty, the POST provides the weighted distribution of the possible SL at each given position. This information is particularly useful for the structural regions where the encoding is difficult or variable. It could be more representative of the 3D structure than the MAP; 3) for better interpretation, the NEFF of SL at each given position is also derived from the entropy of POST. This NEFF is typically close to 1 when the encoding is highly certain and can reach 27 for totally uncertain positions. Therefore this NEFF provides a convenient measurement of the encoding certainty.

**Fig 6:**
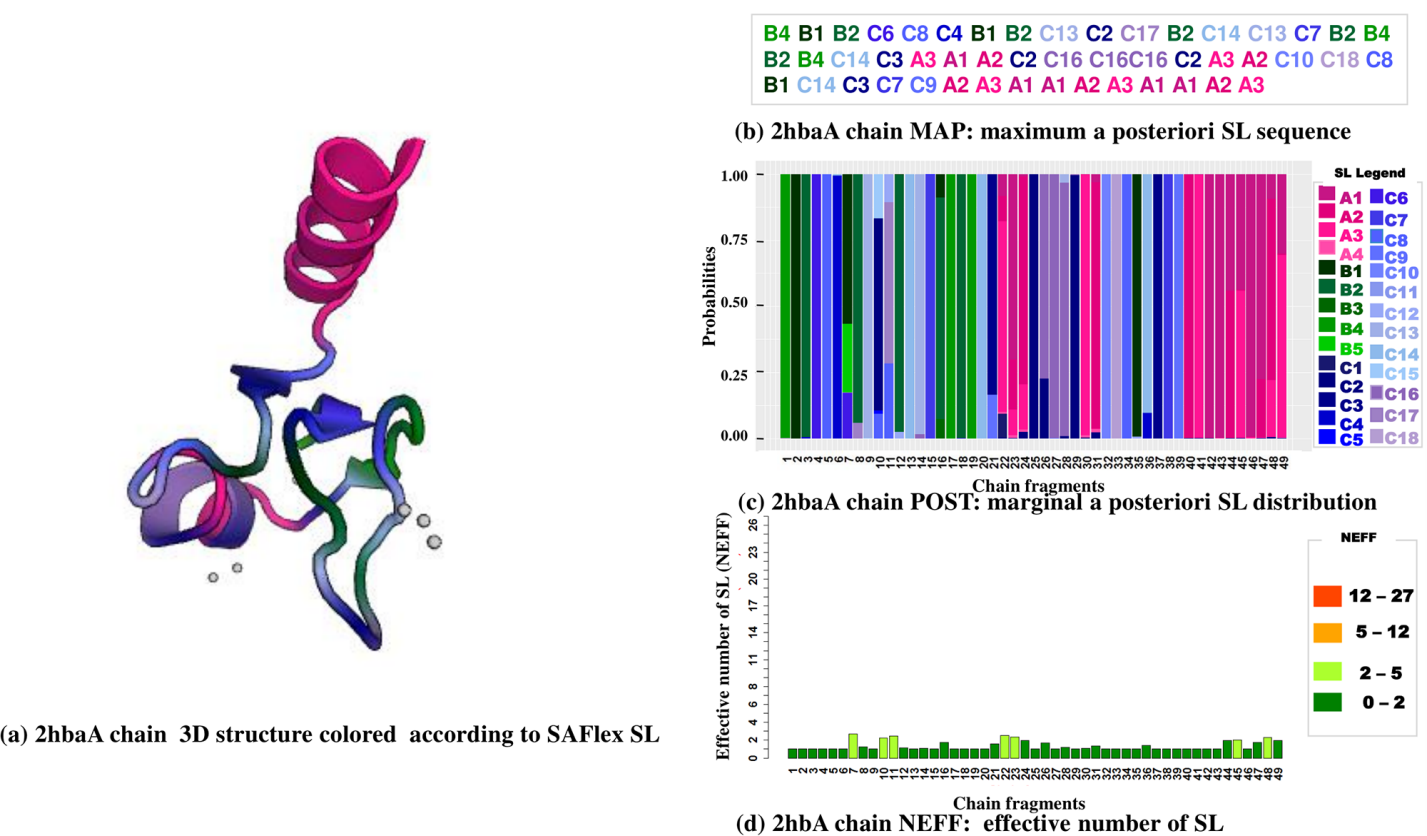
**SAFlex encoding of the 2hba pdb structure, corresponding to the N-terminal domain of the ribosomal protein L9 (NTL9)**: (a) the 2hba 3D structure itself is represented, colored according to the 27 SAFlex SL, (b) the 2hba corresponding SAFlex MAP, (c) the 2hba POST encoding colored according to the 27 SAFlex SL and (d) the 2hba NEFF values.

We can see the 3D chain A of the 2hba PDB and the three corresponding outputs of SAFlex in Fig 6. This structure corresponds to the N-terminal domain of the ribosomal protein L9, (NTL9), a small alpha-beta protein of 52-residue mixed protein. NTL9 has been widely used as a model system for experimental and computational studies of protein folding and for investigations of the unfolded state [54]. In the left panel of Fig 6, the 3D structure of 52 residues is represented, which gives 49 overlapping structural fragments colored according to the 27 SL encoding. In the right panel, MAP, POST and NEFFare represented from top to bottom. The MAP clearly corresponds to a alpha-beta mixed protein with few coil links. In the POST, we can see that most encoding positions are highly certain, even if a few positions display some uncertainty. We observe on the protein three regions of relative uncertainty in fragment positions [7-11], [21-24], and the end region [44-49]. For example, Fragment 10 posterior distribution highlights three possible coil letters: C2 (prob = 0.726), C15 (prob = 0.167), C8 (prob = 0.093), associated with a NEFF = 2.244. Finally, the NEFF graph provides a representation of the encoding uncertainty at each position.

One of the interesting features of structural alphabet encoding is that, by design, it accounts only for the local 3D structure. Similarities between structural sequences (ex: using local or global pairwise alignment with suitable parameters) might then highlight local 3D similarities that global RMSD comparisons might totally miss (ex: two proteins with two very similar domains but different torsion between them). Nevertheless, the calibration of SL similarity score and gap cost for 3D structure comparison is its own research subject for a forthcoming publication.

This uncertainty could typically come from the presence of missing data (see Section “Missing data”) and/or of multiple chains (see Section “Multi-chains”). However, we might observe some encoding uncertainty even in the situation of a single chain for a given protein and no missing data (as in Fig 6). This could be due to several reasons: 1) poor crystallographic quality; 2) properties of the protein such as disordered [55] or flexibility; 3) the fragment might be compatible with several SL due to the training limitations of the SA. However, since our SA is only an approximation of the structure composition of proteins, it is therefore not surprising that some ambiguity remains regardless of the accuracy of the SA. Therefore, this intrinsically leads to a posterior probabilistic distribution of encoding trajectories which is the precise purpose of the “Protein encoding” section.

### Missing data

Many structures in the PDB have missing parts. For example, a representative data set, PDBselect [56], includes 8,565 PDB files with 75% of chains faced with a problem of missing data: either missing coordinates of the complete residues or alpha-carbon atoms.

As explained in the “Materials and Methods” section, our new model rigorously takes into account missing information through probabilistic computations, which depend on the missing pattern of descriptors at the corresponding positions. When descriptors are missing, the local likelihood can still be computed using the marginal Gaussian emission probabilities and this partial information can be combined with the local context to provide some estimations. When only few descriptors are missing, the local likelihood can still be computed using the marginal Gaussian emission probabilities and this partial information can be combined with the local context to provide more reliable estimations in terms of exact SL. When all four descriptors are unavailable, the position is totally uninformative (local likelihood of 1.0 for all SL) but the context of the position is still accounted thanks to the Markov dependence of the model through the transition matrix.

**Fig 7:**
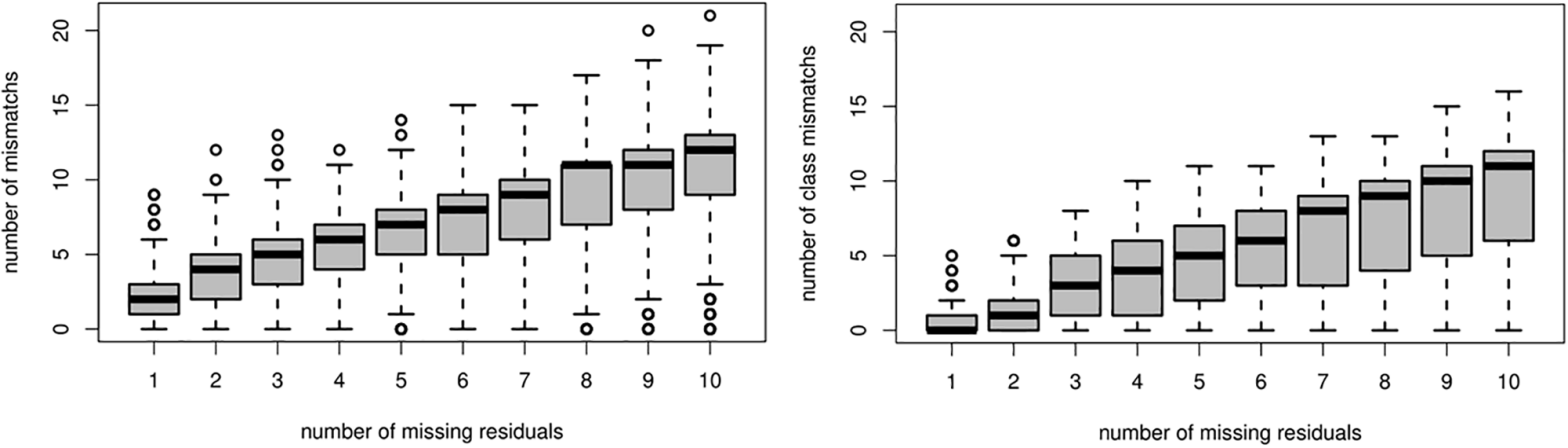
**Average mismatches induced by missing residuals on 10,000 simulations**: average number of SL mismatches (left) and secondary class (helices, beta-strands, coils) mismatches (right) as a function of the number of consecutive missing residues.

In order to quantify the number of SL mismatches induced by the presence of missing residues in the chain in the case of MAP encoding, we designed the following numerical experiment. First, we selected a total of 39 PDB chains (The 39 PDB ids: 1XMK 1MZ9 1QSA 3IIS 1GXM 4K12 4HI8 4DEQ 1UUN 4GV5 2AYD 2O9S 2EVB 3WJT 2E5Y 4A02 3NBC 2DPF 1VMO 3DCL 3C7X 1TL2 1PJX 2XDW 1W6S 1M8N 4E2V 1IGD 1EWF 4BEU 1VBM 2HBA 1A9X 1DL5 1HQ0 1UD9 2CI1 1J0P 1V54) with no missing residue and representative of the variability of available structures in terms of secondary structure types. At most, we used one chain of mainly alpha, mainly beta, alpha-beta or with few regular secondary structure proteins. Then we randomly selected (uniformly) one of these chains, removed the residue information for *k* consecutive residues (random uniform position) with *k* ∈ {1, 2, …, 10}, and then compared the original SL encoding (using the MAP) to the encoding obtained with the chain with missing residues. The experiment was repeated 10, 000 times. The number of SL mismatches and class mismatches (helices, beta-strands, coils) are reported in Fig 7.

Not suprisinsgly, we see that both the mismatches (left) and class-mismatches (right) increase with the number of (consecutive) missing residues in a roughly linear tendency and that we obtain fewer class-mismatches than SL mismatches. We can note one unique missing residue impacts four consecutive fragments, due to their overlapping on three alpha carbons (corresponding to one to three missing descriptors). Quantitatively, for *k* = 1 (resp. *k* = 2) missing residues, we obtain an average of 1.872 SL mismatches, 1st quartile 1, median 2, 3rd quartile 3 (resp. 3.308 with 1st quartile 2, median 4, 3rd quartile 5). In terms of secondary structure information, for *k* = 1 (resp. *k* = 2) missing residues, we obtain a weak average of 0.479 secondary structure SL mismatches, 1st quartile 0, median 0, 3rd quartile 1 (resp. and 1.127, 1st quartile 0, median 1, 3rd quartile 2. These figures are small compared to the average length of 199.3 SL, (1st quartile 67.0, median 151.0, 3rd quartile 317.0) of the 10, 000 considered chains. Unsurprisingly, the prediction of the secondary structure is more robust to missing data than the SL prediction itself.

**Fig 8:**
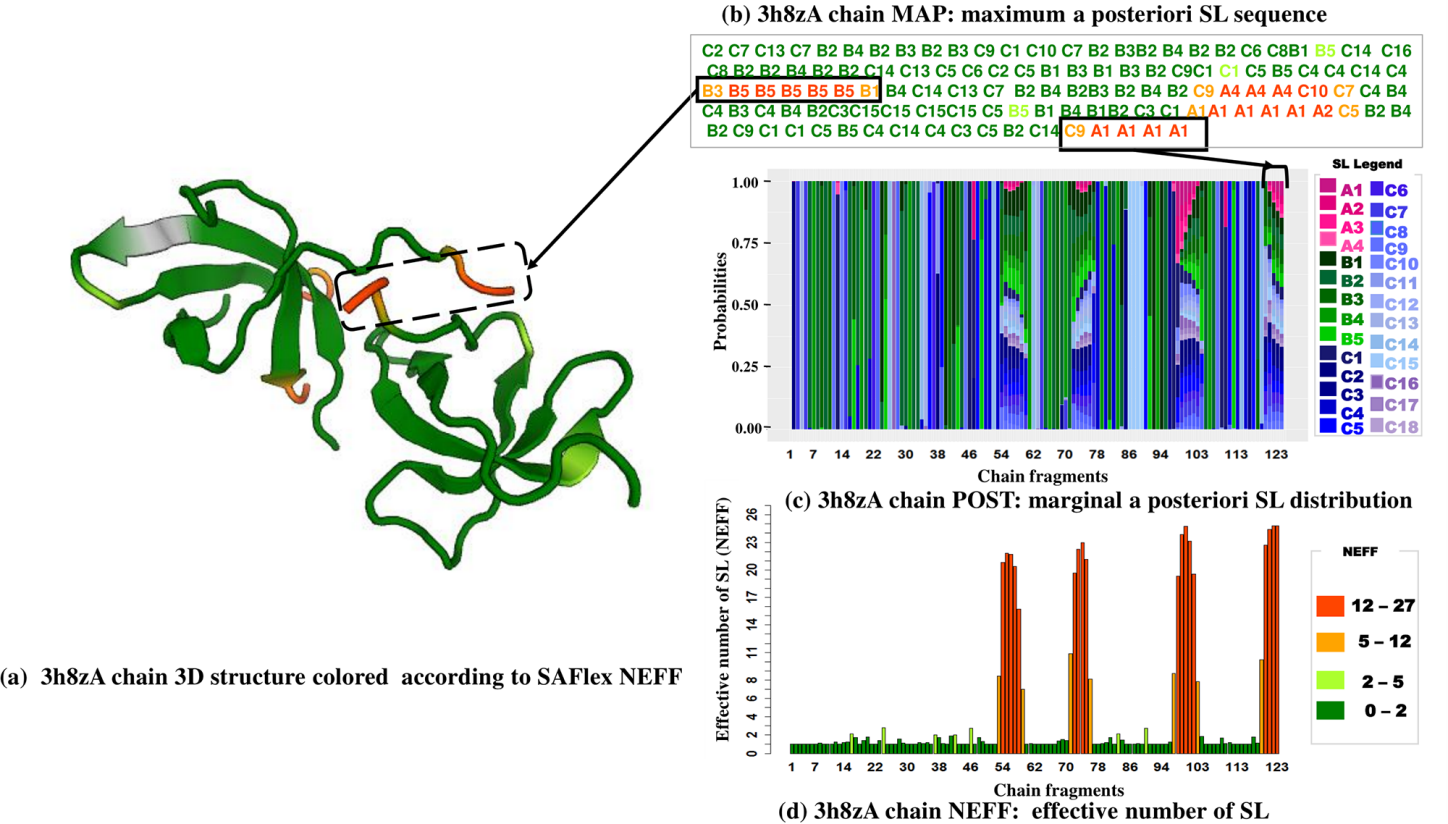
**Missing conformation detection of the 3h8z pdb entry, corresponding to the homo sapiens fragile X mental retardation syndrome-related protein 2 associated with FXR2 gene**: (a) the 3h8z 3D structure is presented colored according to the NEFF value legend, (b) the 3h8z corresponding SAFlex MAP colored according to the NEFF value legend, (c) the 3h8Z POST encoding and (d) the 3h8z NEFF values.

Finally, this confirms the resulting encoding is highly uncertain when repeated missing residues appear in a given region of the 3D structure, but interestingly this information is provided by SA-Flex by high NEFF values. To illustrate the interest of different encoding proposed by SAFlex, in case of missing data, we consider the protein 3h8z corresponding to the homo sapiens fragile X mental retardation syndrome-related protein 2 associated with FXR2 gene. The FMRP, FXR1 and FXR2 proteins comprise a small family of highly conserved proteins that appear to be important in translational regulation, particularly in neuronal cells [57]. In Fig 8, we can see the crystal structure of the tudor domains from FXR2 (left panel) from the PDB 3h8z and the SAFlex outputs from top to bottom: MAP, POST, and NEFF (right panel). This 3D structure corresponds to 123 overlapping structural fragments colored according to their NEFF values. In Fig 8(a) and 8(b), we observe that a big part of the protein correspond to beta-strand SL linked by short coil SL regions, associated with weak uncertainties, as illustrated in Fig 8(c) and 8(d). This is coherent with the fact Tud1 domain forms a canonical tudor barre comprising five highly twisted antiparallel beta-strands. However, we clearly observe the presence of four regions of high uncertainty (NEFF close to 27). These four regions correspond to the 16 residues with missing 3D coordinates information in the PDB file with the first region [53-59] predicted as disorder using psipred website [58, 59]. Despite this high uncertainty, these positions being associated to a NEFF close to 27, one should note that the MAP suggests encodings for these four regions. This further illustrates the interest of the multiple outputs of SAFlex.

### Multi-chains

**Fig 9:**
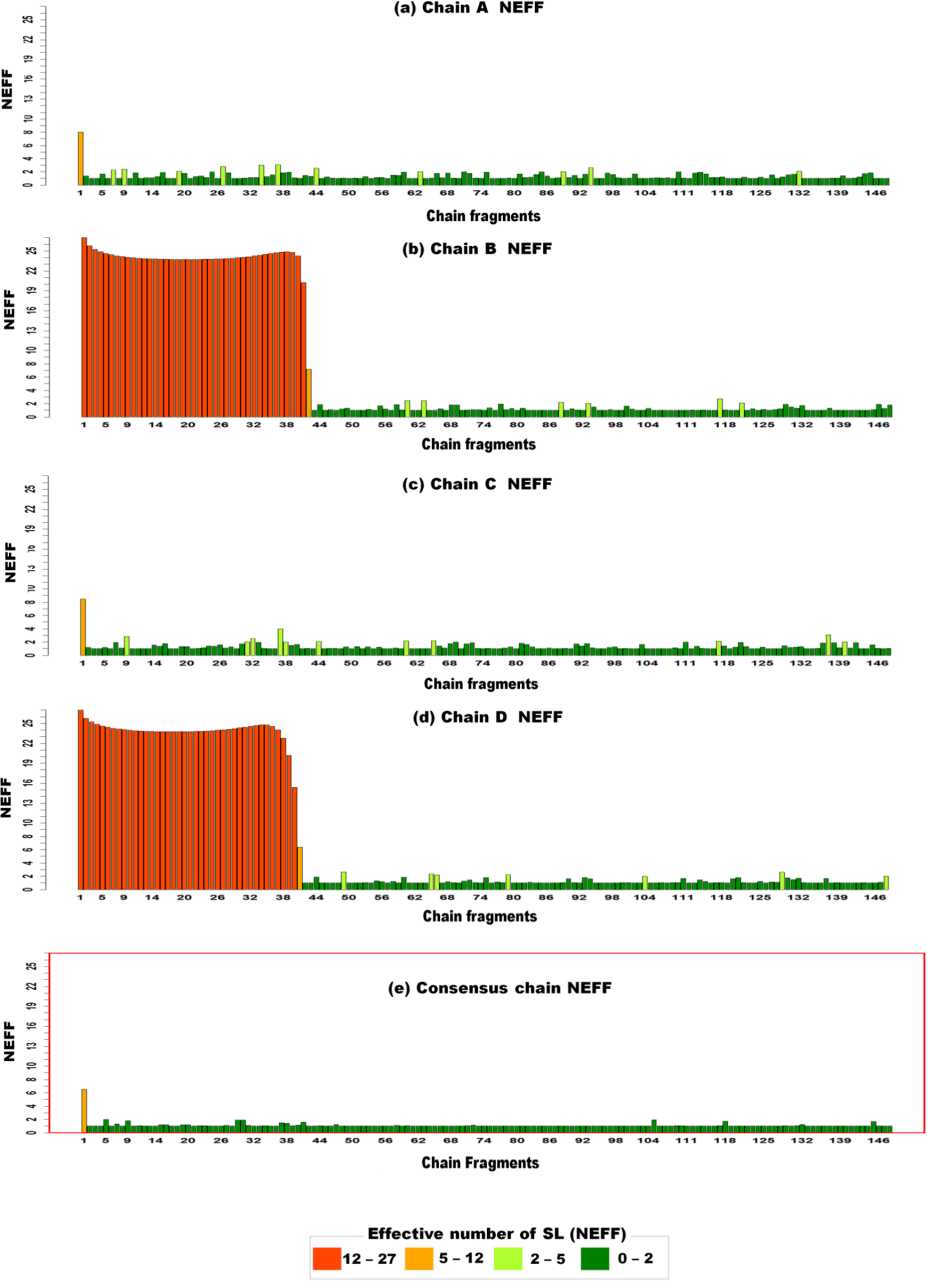
NEFF of the four monomers and consensus chain from the 1gme pdb, corresponding to the small Heat Shock Proteins (sHSPs). The x-axis of the charts correspond to the 148 fragment numbers and the y-axis to the NEFF values, from 1 to 27. Each bar is colored according to the NEFF value legend. The charts **(a), (b), (c), (d)** correspond to the NEFF values for the four monomer chains A, B, C and D and the chart **(e)**, in a red box to the consensus encoding of the four monomers.

In recent decades, many protein complex structures containing multiple chains have been determined: homomers, heteromers or different PDB files corresponding to the same protein in different conditions. If heteromers are naturally encoded as different structural sequences, homomers can be considered as replicates of the same underlying structure. SAFlex proposes to encode homomers either as independent structures (one structural sequence per chain) or as a single consensus one, where a single hidden structural sequence is shared by all homomeric chains. The resulting consensus encoding hence represents the variability of the homomer across the chains. This variability is either due to measurement uncertainty or to intrinsic flexibility. One illustration is presented on the small Heat Shock Proteins (sHSPs), which are important in stress tolerance and play an essential role in preventing aggregation of target proteins [60]. They participate in protecting, maintaining and regulating specific protein functions. The PDB entry 1gme corresponds to HSP16.9, a member of the sHSPs, that assembles into a dodecameric double disk. In the PDB file, an available tetramer can be used to reconstruct the dodecamer by symmetry operations [61]. The monomer’s residue length is 151 which gives 148 overlapping structural fragments. The four monomers have a global common structure, called the alpha crystallin domain signature [60] but have differences in some regions: the 42 N-terminal residues are missing in the two monomers B and D, whereas the N-terminal arm in A and C monomers is fully resolved and composed of helices connected by random coils. In Fig 9, we can see the values of NEFF for the four chains (upper panel) and for the consensus encoding (lower panel). The two missing regions of chains B and D are clearly highlighted (NEFF close to 27 on positions [1, 42]). The overall uncertainty of encoding along the four chains is quite large with an average value of NEFF ≃ 4.4 on all the regions and of NEFF ≃ 1.3 when excluding missing regions. In the lower panel, we can see that the consensus encoding on all regions has a much lower uncertainty of NEFF ≃ 1.1. This illustrates the interest of the consensus approach which not only provides an encoding for the complete protein despite the missing patterns of chains B and D, while taking advantage of the replicates to refine the structural encoding.

**Fig 10:**
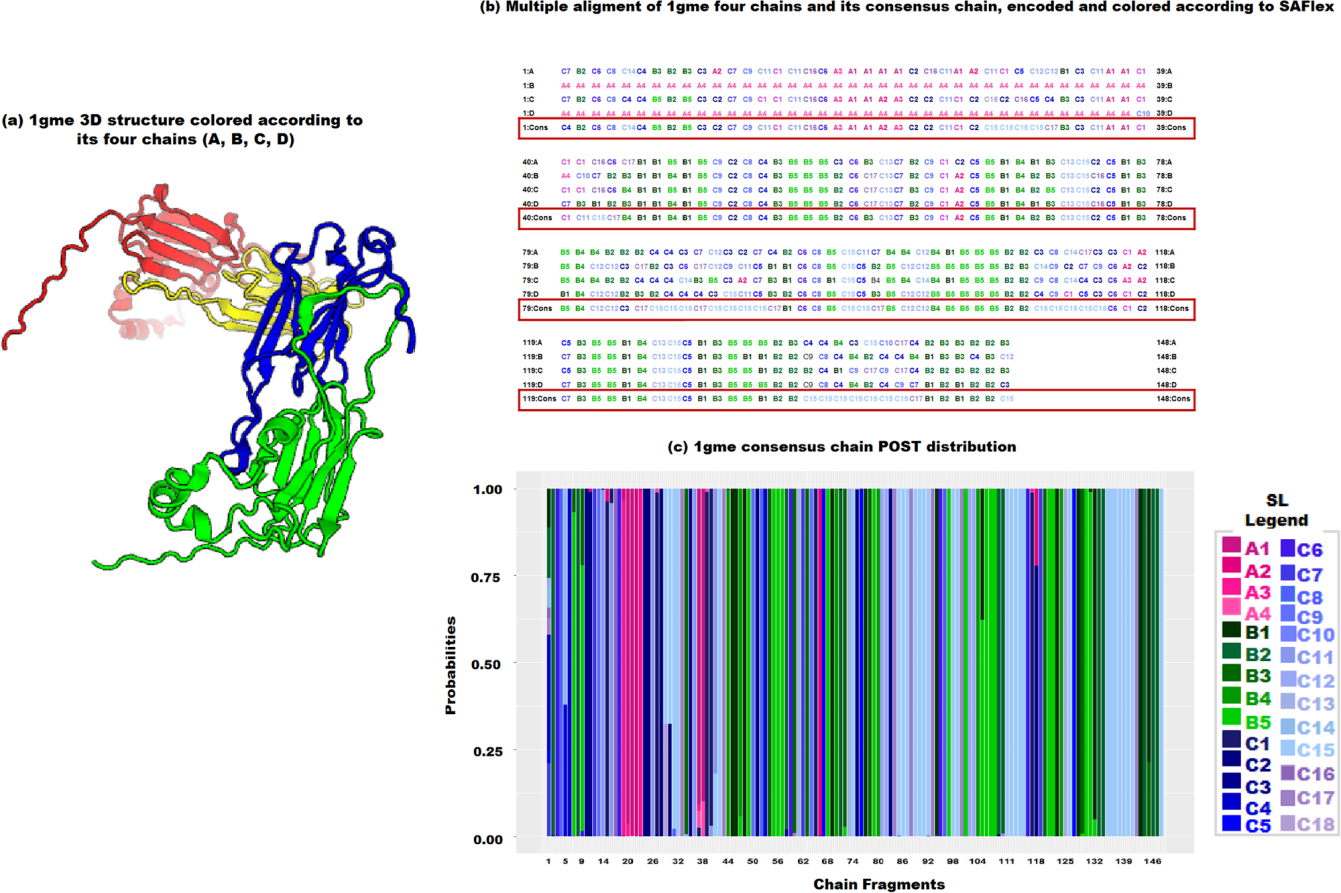
**SAFlex independent and consensus encoding of the 1gme four chains**: (a) the 3D structure of 1gme is displayed by textcolorredPV, TO DEFINE, colored according to its four chains: A in red, B in yellow, C in green and D in blue. Each monomer corresponds to 148 overlapping structural fragments; (b) the multiple alignment of the MAP of the four encoded chains and the consensus encoding in a red box. Each letter is colored according to SAFlex SL; (c) the POST encoding for the consensus chain. The x-axis corresponds to the fragment numbers and the y-axis represents the posterior probabilities.

In Fig 10, we see (a) the 3D structure of the four chains of 1gme (left panel), (b) the multiple alignment of the MAP for the four chains and the consensus encoding as well as (c) the POST encoding for the consensus encoding. The missing regions in chains B and D appear clearly in the MAP with long runs of the SL-A4; this is coherent in the absence of any additional information since this structural letter has the highest probability of self-transition in the SA (see transition matrix in the supplementary data). However, chains A and C provide informative MAPs for the corresponding positions and the result is clearly consistent with the consensus’ MAP. For most of the remaining positions, we observe a strong concordence among all encodings reflecting low structural variability. However, for some regions in fragment positions [29-32], [85-92], [110-116] and [136-142], there is higher variability across the MAP of the fours chains, indicated by different SL for the four chains. This suggests that some of these positions could correspond to intrinsic flexible chain positions or to resolution uncertainties. In this context, the consensus encoding tries to find the most adequate common structural letter to reflect this variability and selects the SL-C15 (former F in HMM-SA) which is known to correspond to the fuzzy coil state of the alphabet [40] and is associated with very high posterior probabilities (close to 1), Fig 10(c). This result is clearly consistent with the assumption of a common underlying structure among the different chains. However, it also shows its limits in case of conformation plasticity. In this case, the independent chain encodings can be carrefully explored to detect variable positions and SL changes potentially due to intrinsic flexilibity, to partner binding or to sequence mutation effects [25].

## Conclusion

Conceiving protein structures has moved beyond static representations to include dynamic aspects of quaternary structures, like conformational changes upon binding and structural fluctuations occurring within fully assembled complexes [38].

SA encodings are known to perform well for fine study of their structural properties. However, SA have to be improved to better understand uncertainties such as missing data and intrinsic flexibility observed between different available replicates in the PDB. This has an impact on our knowledge of protein functions and their disordered regions, which contribute to the protein capacity to establish interactions with different partners.

In this paper, we presented SAFlex, extended from HMM-SA (with similar fragment descriptors, number of letters (27), Gaussian distribution per letter, and transition matrix) to provide structure encoding in terms of missing residues as well as uncertainties. SAFlex has the following three main novelties: 1) allows for three different encoding outputs (MAP, marginal posterior distribution, and entropy-related statistics); 2) new implementation is robust for any missing data pattern in the PDB file; and 3) new model can take into account replicates and include a new consensus encoding for homomers. These correspond to important improvements as there are many chains with missing data (e.g. 75% of PDBselect chains faced with a problem of missing data [56]) and most of available structural information on protein complexes concerns homomers [36]. All of these improvements are freely available to the public through a web server application.

Concerning missing data, as pointed out by our experiments, when there are too many consecutive missing residues, the loss of information eventually leads to many encoding errors. It could hence be interesting to include primary or secondary structures into the alphabet in order to exploit this additional information in the context of large portion of missing residues in the PDB chains.

Another interesting features of our structural alphabet encoding is that, by design, it accounts only for the local 3D structure. Similarities between structural sequences (ex: using local or global pairwise alignment with suitable parameters) might then highlight local 3D similarities that global RMSD comparisons might totally miss (ex: two proteins with two very similar domains but different torsion between them). Nevertheless, the calibration of SL similarity score and gap cost for 3D structure comparison is its own research subject for a forthcoming publication.

Having implemented a basic version of SAFlex, our next step would be to use up-to-date large scale data to train a new SA with the following characteristics: more parsimony with uniform starting distribution, model selection using penalized approaches (e.g. adaptive ridge [62]) for the (very sparse) transition matrix, more and/or different descriptors to ensure a bijection between the 3D fragment conformation and the descriptor space, and model extensions to allow for multi-conformations in addition to the multi-chains feature.

## Supporting information

S1 TableThe table contains the Gaussian emission parameter of SAFlex 27.Rows correspond to the SAFlex SL. The SL are classified by group: alpha-helices, coils and beta-strands. For each structural letter is given the four descriptors $X=\left(X^{1},X^{2},X^{3},X^{4}\right)\in R^{4}$ and the covariance matrix Σ. Rows and colons of the matrix corresponds to descriptors.(TEX)

## Abbreviations

ENT: marginal posterior entropy
HMM: hidden Markov model
MAP: Maximum a Posteriori
NEFF: effective number of structural letters
PDB: Protein Data Bank
POST: marginal posterior distribution
SA: structural alphabet
SL: structural letters
1D: one-dimensional
3D: three-dimensional
